# 24(*S*)-Hydroxycholesterol induces ER dysfunction-mediated unconventional cell death

**DOI:** 10.1038/s41420-019-0192-4

**Published:** 2019-07-05

**Authors:** Yasuomi Urano, Diep-Khanh Ho Vo, Araki Hirofumi, Noriko Noguchi

**Affiliations:** Department of Medical Life Systems, Faculty of Life and Medical Sciences, Doshisha University, Kyoto, 610-0394 Japan

**Keywords:** Apoptosis, Sterols

## Abstract

Endoplasmic reticulum (ER) stress induced by disruption of protein folding activates the unfolded protein response (UPR), which while generally pro-survival in effect can also induce cell death under severe ER stress. 24(*S*)-hydroxycholesterol (24S-OHC), which is enzymatically produced in the ER of neurons, plays an important role in maintaining brain cholesterol homeostasis but also shows neurotoxicity when subjected to esterification by acyl-CoA:cholesterol acyltransferase 1 (ACAT1) in the ER. In this study, we demonstrated that the accumulation of 24S-OHC esters in human neuroblastoma SH-SY5Y cells evoked the UPR with substantially no pro-survival adaptive response but with significant activation of pro-death UPR signaling via regulated IRE1-dependent decay (RIDD). We further found that accumulation of 24S-OHC esters caused disruption of ER membrane integrity and release of ER luminal proteins into cytosol. We also found that de novo synthesis of global proteins was robustly suppressed in 24S-OHC-treated cells. Collectively, these results show that ER dysfunction and the accompanying RIDD-mediated pro-death UPR signaling and global protein synthesis inhibition are responsible for 24S-OHC ester-induced unconventional cell death.

## Introduction

Upon accumulation of unfolded/misfolded proteins in the endoplasmic reticulum (ER)—a condition referred to as ER stress—cells activate an adaptive response—this response being referred to as the unfolded protein response (UPR)—to restore ER homeostasis and maintain the fidelity of protein-folding^1^. In mammalian cells, the UPR comprises three major signaling pathways, which are respectively mediated by inositol-requiring enzyme 1 (IRE1), protein kinase RNA-like ER kinase (PERK), and activating transcription factor 6 (ATF6). The UPR processes include induction of ER chaperones, inhibition of protein synthesis, and stimulation of retrograde transport of misfolded proteins from the ER into the cytosol for ubiquitination and destruction by ER-associated degradation (ERAD)^2^. However, under unresolvable ER stress conditions, the UPR represses the adaptive response and triggers cell death^3–6^.

On activation of IRE1, phosphorylated IRE1 becomes an active endoribonuclease (RNase) that cleaves an intron of *X-box binding protein 1* (*XBP1*) mRNA, resulting in the translation of bZIP-containing transcription factor XBP1s^1^. XBP1s induces the expression of several genes involved in the UPR to activate pro-survival mechanisms. IRE1 RNase activity also contributes to post-transcriptional degradation of a subset of ER-localized mRNAs through a process known as regulated IRE1-dependent decay (RIDD)^7–9^. Although RIDD may help to reduce the folding load of nascent proteins and alleviate ER stress as a pro-survival mechanism, continuous decay of RIDD substrates by unmitigated ER stress leads to pro-death outputs^4,10^. Activated IRE1 also promotes apoptosis through the activation of apoptosis signal-regulated kinase 1 (ASK1; also known as MAP3K5), JUN N-terminal kinase (JNK), and p38 MAPK. Activation of PERK by autophosphorylation induces pro-survival signaling and pro-apoptotic signaling. Activated PERK phosphorylates eukaryotic translation initiator factor 2α (eIF2α) and causes general translational attenuation which is a pro-survival process, as it reduces the number of proteins entering the ER^11^. In addition, phosphorylation of eIF2α also induces the specific translation of ATF4. ATF4 controls the expression of genes that encode not only cytoprotective proteins but also pro-apoptotic proteins, including C/EBP-homologous protein (CHOP)^12^. ATF6 is a type II transmembrane protein containing bZIP transcription factor in its cytosolic domain. Upon occurrence of ER stress, ATF6 translocates to the Golgi apparatus, where it is cleaved by site-1 protease and site-2 protease. The cleaved cytosolic domain of ATF6 activates transcription of target genes such as ER chaperones. There is growing evidence that not only accumulation of unfolded/misfolded proteins in the ER lumen, but also alterations in membrane lipid desaturation and aberrant phospholipid composition may also be potent in activating the UPR^13,14^. There is, however, little known at present about whether oxidation products of lipids might activate the UPR.

24(*S*)-hydroxycholesterol (24S-OHC) is one of the enzymatic oxidation products of cholesterol and plays an important role in maintaining brain cholesterol homeostasis^15–18^. While 24S-OHC has important physiological and protective functions in the brain, several lines of evidence suggest that dysregulation of 24S-OHC metabolism in the brain may contribute to the development of neurodegenerative diseases such as Alzheimer’s disease (AD) and Parkinson’s disease (PD)^19–26^. Furthermore, we and other groups have shown that 24S-OHC possesses a potent neurotoxicity that may be involved in the etiology of neurodegenerative disease^27,28^. We have previously demonstrated that 24S-OHC elicits caspase-independent cell death in human neuroblastoma SH-SY5Y cells, and rat primary cortical neuronal cells not expressing caspase-8^28^. 24S-OHC-induced cell death is partially but significantly suppressed by Necrostatin-1 (Nec-1), an inhibitor of receptor interacting serine/threonine kinase 1 (RIPK1) or knockdown of RIPK1. However, as necroptosis is a form of regulated necrotic cell death that depends on MLKL, RIPK3, and (at least in some settings) on the kinase activity of RIPK1^29^, and since we subsequently showed that RIPK1, but neither RIPK3 nor MLKL, is expressed in SH-SY5Y cells^30^, we considered that the type of cell death induced by 24S-OHC in SH-SY5Y cells is necroptosis-like.

Acyl-CoA:cholesterol acyltransferase 1 (ACAT1), an ER-resident enzyme, catalyzes the esterification of free cholesterol to form cholesteryl esters^31^. We found that ACAT1 also utilizes 24S-OHC and long-chain unsaturated fatty acid as substrates to form 24S-OHC ester^32,33^. We further found that accumulation of 24S-OHC esters leads to formation of atypical lipid droplet (LD)-like structures coupled with an enlarged membrane structure that appears to be a swollen ER structure^33^. Since ACAT inhibitor or ACAT1 siRNA suppresses both 24S-OHC-induced LD-like structure formation and cell death, we concluded that ACAT1-catalyzed 24S-OHC esterification and formation of atypical LD-like structures having abnormal ER morphology are initial key events in 24S-OHC-induced cell death; however, the specific mechanism by which 24S-OHC esterification activates cell death signaling still remains to be elucidated.

In this study, we found that esterification of 24S-OHC evoked the UPR but the downstream pro-survival adaptive response was not substantially activated, finding instead that RIDD is implicated in 24S-OHC-induced cell death. We also demonstrate that accumulation of 24S-OHC esters caused disruption of ER membrane integrity, resulting in the release of a number of ER-resident proteins into cytosol and the suppression of de novo protein synthesis. Taken together, we conclude that ER dysfunction, and the accompanying pro-death UPR signaling and protein synthesis inhibition, are responsible for cell death induced by ACAT1-catalized 24S-OHC esterification.

## Results

### Accumulation of 24S-OHC esters activated the UPR in SH-SY5Y cells

**Fig. 1:**
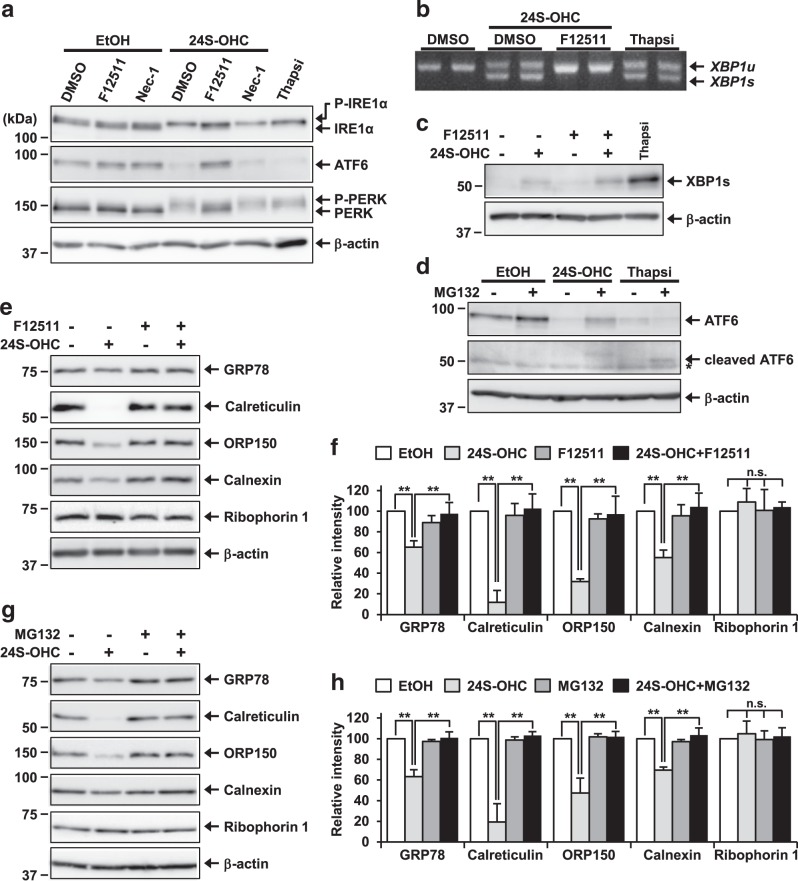
Accumulation of 24S-OHC esters activated the UPR signaling pathway and downregulated expression of ER chaperone proteins in SH-SY5Y cells. **a**–**c** SH-SY5Y cells were pretreated with 5 μM F12511 for 15 min or with 100 μM Nec-1 (**a**) for 1 h and then exposed to 50 μM 24S-OHC for 6 h. Cells were also treated with 3 μM thapsigargin (Thapsi) for 3 h. **a** Whole-cell lysates were subjected to immunoblotting with appropriate antibodies as indicated. **b** The unspliced (*XBP1u*) and spliced (*XBP1s*) *XBP1* mRNAs were analyzed by RT-PCR. **c** Whole-cell lysates were immunoblotted with antibodies specific for XBP1s or β-actin. **d** Cells were pretreated with 20 μM MG132 for 30 min and then exposed to 50 μM 24S-OHC or 3 μM thapsigargin for 6 h. Whole-cell lysates were immunoblotted with antibodies specific for ATF6 or β-actin. Asterisks denote nonspecific bands. **e**, **f** Cells were treated as in panel **a**. **g**, **h** Cells were treated as in panel **d**. **e**, **g** Whole-cell lysates were subjected to immunoblotting with appropriate antibodies as indicated. **f**, **h** Band intensities were quantified by densitometric scanning, relative intensity is shown. Mean ± SD *n* = 3, **P < 0.01, n.s. not significant

To investigate the activation of UPR signaling by 24S-OHC, we first examined IRE1 autophosphorylation by immunoblotting. In a control experiment, the well-established ER stress inducer thapsigargin, which inhibits ER Ca^2+^-ATPase, induced phosphorylation of IRE1α as confirmed by observation of mobility shift of the IRE1α band in SDS-PAGE as compared with vehicle condition in SH-SY5Y cells (Fig. 1a). The same shift in the IRE1α band was observed in cells treated with 50 μM 24S-OHC for 6 h, suggesting that 24S-OHC treatment induced IRE1α phosphorylation. 24S-OHC-induced IRE1α phosphorylation was inhibited by cotreatment with F12511 but not by Nec-1 (Fig. 1a). We further found that *XBP1* splicing was induced in cells treated with 24S-OHC in similar fashion as was observed with thapsigargin treatment (Fig. 1b). 24S-OHC-induced *XBP1* splicing was also inhibited by cotreatment with F12511. However, XBP1s protein expression was less remarkably induced in 24S-OHC-treated cells than in thapsigargin-treated cells (Fig. 1c), suggesting that IRE1α was activated but that downstream XBP1s was not substantially activated in 24S-OHC-treated cells.

We also found that there was phosphorylation of PERK as demonstrated by the occurrence of mobility shift and reduction in the amount of full-length ATF6, which occurred in cells treated with 24S-OHC in similar fashion as was observed with thapsigargin treatment (Fig. 1a). Similar to our observations with respect to IRE1α, these changes were found to be suppressed by cotreatment with F12511, but not by Nec-1. Because the cleaved form of ATF6 is relatively unstable due to proteasomal degradation^34^, we also examined activation of ATF6 in the presence of the proteasome inhibitor MG132, finding that the cleaved ATF6 was observed in cells treated with thapsigargin in the presence of MG132, but that uncleaved full-length ATF6 was observed in cells treated with 24S-OHC (Fig. 1d), suggesting that 24S-OHC-induced reduction of full-length ATF6 was not due to processing, but was instead due to proteasomal degradation.

We further found that expression of ER chaperone proteins (glucose-regulated protein 78 kDa (BiP/GRP78), calreticulin, ORP150, and calnexin) but not ribophorin 1 was reduced in 24S-OHC-treated cells, and that this reduction was suppressed by cotreatment with F12511 (Fig. 1e, f). We also found that this reduction was inhibited by cotreatment with MG132 (Fig. 1g, h), suggesting that 24S-OHC-induced downregulation of ER chaperone proteins was due to proteasomal degradation. Time-course experiments revealed that IRE1α phosphorylation occurred substantially, simultaneously with reduction in the amount of calreticulin (Fig. S1). Collectively, these data indicate that ACAT1-mediated 24S-OHC esterification induces ER stress, leading to activation of UPR signaling pathways, but that pro-survival mechanisms are not substantially activated in SH-SY5Y cells.

### Upregulation of CHOP expression was not involved in 24S-OHC-induced cell death in SH-SY5Y cells

**Fig. 2:**
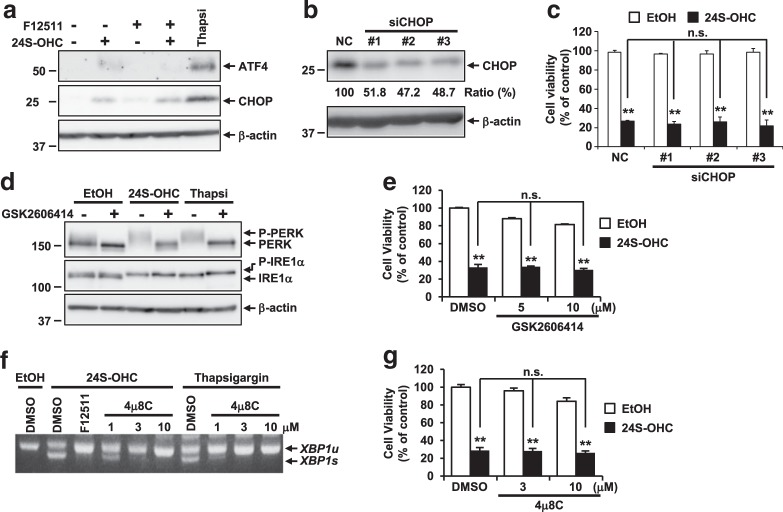
PERK and IRE1-XBP1 axes were not involved in 24S-OHC-induced cell death in SH-SY5Y cells. **a** SH-SY5Y cells were treated as in Fig. 1a. Whole-cell lysates were immunoblotted with antibodies specific for ATF4, CHOP, or β-actin. **b**, **c** Cells were transfected with CHOP (siCHOP #1-#3) or negative control (NC) siRNA oligo for 24 h. **b** Whole-cell lysates were immunoblotted with antibodies specific for CHOP or β-actin. Relative expression levels of CHOP are shown. **c** The cells were exposed to 50 μM 24S-OHC for 24 h. Cell viability was measured by LDH assay. **P < 0.01, when compared with cells treated with vehicle or among 24S-OHC-treated groups. **d** Cells were pretreated with 5 μM GSK2606414 for 30 min then exposed to 50 μM 24S-OHC or 3 μM thapsigargin for 6 h. Whole-cell lysates were immunoblotted with antibodies specific for PERK, IRE1α, or β-actin. **e** Cells were pretreated with 5 or 10 μM GSK2606414 for 30 min then exposed to 50 μM 24S-OHC for 24 h. Cell viability was measured by WST-8 assay. **P < 0.01. **f** Cells were pretreated with 5 μM F12511 for 15 min or with 1–10 μM 4μ8 C for 1 h then exposed to 50 μM 24S-OHC for 6 h or 1 μM thapsigargin for 3 h, respectively. *XBP1* mRNAs were analyzed by RT-PCR. **g** Cells were pretreated with 3 or 10 μM 4μ8 C for 1 h and then exposed to 50 μM 24S-OHC for 24 h. Cell viability was measured by WST-8 assay. **P < 0.01

To investigate whether activation of pro-death UPR signaling was implicated in 24S-OHC-induced cell death, we first examined the induction of ATF4 and CHOP in SH-SY5Y cells, the results showing that ATF4 and CHOP expression was only moderately induced in 24S-OHC-treated cells as compared with the more pronounced expression, thereof observed in thapsigargin-treated cells (Fig. 2a). Furthermore, when 24S-OHC-induced CHOP upregulation was suppressed by CHOP siRNA (Fig. 2b), we found that cell death was not suppressed (Fig. 2c), suggesting that CHOP induction is not involved in the 24S-OHC-induced cell death machinery.

### PERK, IRE1-XBP1, and IRE1-ASK1 axes were not involved in 24S-OHC-induced cell death in SH-SY5Y cells

We then evaluated the effect of the PERK inhibitor GSK2606414, the results showing that GSK2606414 treatment inhibited autophosphorylation of PERK in cells treated with either 24S-OHC or thapsigargin (Fig. 2d). As expected, 24S-OHC-induced IRE1α phosphorylation was not inhibited by GSK2606414 treatment. Under the conditions tested, 24S-OHC-induced cell death was not suppressed by GSK2606414 (Fig. 2e), suggesting that the PERK activation was not involved in 24S-OHC-induced cell death.

We next evaluated the effects of the selective IRE1α RNase inhibitor 8-formyl-7-hydroxy-4-methylcoumarin (4μ8C), results showing that 4μ8 C inhibited *XBP1* splicing in a concentration-dependent manner in cells treated with either 24S-OHC or thapsigargin (Fig. 2f), but that 4μ8C did not inhibit 24S-OHC-induced cell death (Fig. 2g). These results indicate that inhibition of IRE1α-mediated *XBP1* splicing by 4μ8 C did not prevent 24S-OHC-induced cell death in SH-SY5Y cells.

We also evaluated the effect of the selective inhibitor of ASK1 (NQDI-1), p38 (SB203580), or JNK (SP600125) on 24S-OHC-induced cell death, the results showing that neither NQDI-1 nor SB203580 nor SP600125 was able to prevent 24S-OHC-induced cell death (Fig. S2A–C), suggesting that neither ASK1 nor p38 or JNK is implicated in 24S-OHC-induced cell death.

As the small molecular chemical chaperone 4-phenylbutyric acid (4-PBA) was reported to protect against ER stress-mediated neuronal cell death by aiding in protein folding^35,36^, we tested the effects of 4-PBA on 24S-OHC-induced cell death, finding as a result that cotreatment with 1 mM of 4-PBA significantly mitigated thapsigargin-induced cell death, but did not affect 24S-OHC-induced cell death (Fig. S2D), suggesting that the increase in ER folding capacity produced by 4-PBA was ineffective in decreasing 24S-OHC-induced cell death.

### Inhibition of RIDD mitigated 24S-OHC-induced cell death in SH-SY5Y cells

**Fig. 3:**
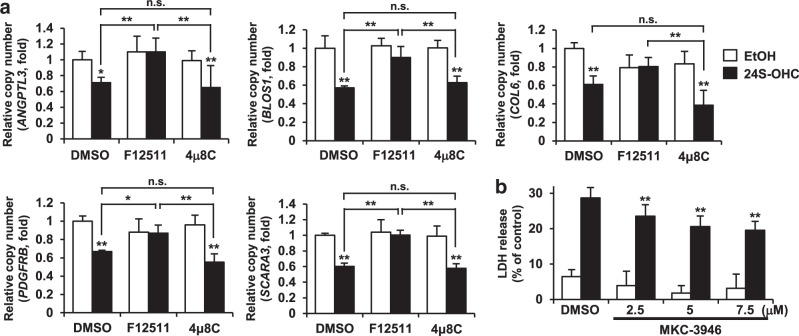
Inhibition of RIDD mitigated 24S-OHC-induced cell death in SH-SY5Y cells. **a** SH-SY5Y cells were pretreated with 5 μM F12511 for 15 min or with 10 μM 4μ8 C for 1 h and then exposed to 50 μM 24S-OHC for 6 h. The mRNA levels of RIDD target genes (*ANGPTL3, BLOS1, COL6, PDGFRB*, and *SCARA3*) were quantified by real-time PCR. Results are shown as normalized to levels measured for *RPL32*. *P < 0.05, **P < 0.01, when compared with cells treated with vehicle or among 24S-OHC-treated groups. **b** Cells were pretreated with 2.5–7.5 μM MKC-3946 for 1 h then exposed to 50 μM 24S-OHC for 24 h. Cell viability was measured by LDH assay. **P < 0.01, when compared with cells treated with 24S-OHC alone

We next investigated whether RIDD was implicated in 24S-OHC-induced cell death. Since RIDD targets multiple mRNA substrates^37–39^, we evaluated the expression levels of a series of RIDD substrates including *ANGPTL3*, *BLOS1*, *COL6*, *PDGFRB*, and *SCARA3*, the results showing that the expression of all genes examined was significantly reduced in cells treated with 24S-OHC for 6 h compared to the vehicle-control condition in SH-SY5Y cells (Fig. 3a), suggesting that 24S-OHC treatment activated the RIDD pathway. Downregulation of all genes tested was inhibited by cotreatment with F12511, indicating that activation of RIDD occurred in response to ACAT1-mediated 24S-OHC esterification.

We then sought to evaluate the involvement of IRE1α-mediated RIDD in 24S-OHC-induced cell death. As it had been reported that knockdown of IRE1α by siRNA induced cell death in SH-SY5Y cells^40^, we therefore first examined the effect of 4μ8c on 24S-OHC-induced downregulation of RIDD substrates. Unlike the inhibitory effect of 4μ8C observed on IRE1α-mediated *XBP1* splicing (Fig. 2f), 10 μM, 4μ8 C did not suppress the downregulation of any gene examined in 24S-OHC-treated cells (Fig. 3a). Although other studies^38^ reported that a high concentration of 4μ8 C is necessary to inhibit RIDD, we found that anything more than 15 μM 4 μ8 C had cytotoxic effect on SH-SY5Y cells (data not shown). We therefore chose another inhibitor of IRE1α RNase activity, i.e., MKC-3946^41^, and found that MKC-3946 significantly inhibited 24S-OHC-induced cell death in a concentration-dependent manner (Fig. 3b). As expected, 7.5 μM MKC-3946 significantly blocked the 24S-OHC-induced downregulation of *BLOS1* and *SCARA3* expression (Fig. S3). Taken together, these results indicated that IRE1α-mediated RIDD plays an important role in the mechanism of 24S-OHC-induced neuronal cell death.

### Accumulation of 24S-OHC esters induced disruption of ER membrane integrity in SH-SY5Y cells

**Fig. 4:**
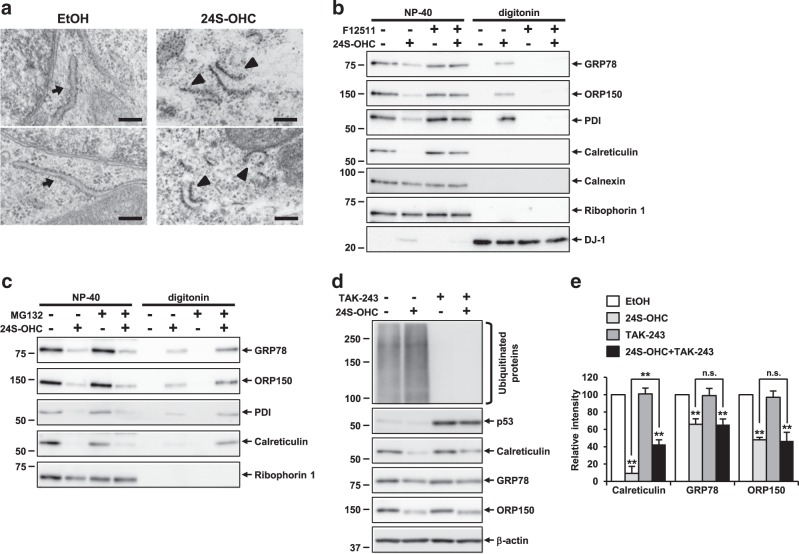
Accumulation of 24S-OHC esters induced disruption of ER membrane integrity, resulting in release of ER luminal proteins into cytosol, in SH-SY5Y cells. **a** SH-SY5Y cells were treated with 50 μM 24S-OHC for 3 h. Cells were subjected to electron microscopy. Representative images are shown. Arrows indicate intact rough ER. Arrowheads indicate broken-membrane ER structures. Scale bar, 0.2 μm. **b**, **c** Cells were pretreated with 5 μM F12511 for 15 min (**b**) or 20 μM MG132 for 30 min (**c**) and then exposed to 50 μM 24S-OHC for 3 h (**b**) or 6 h (**c**). Cells were then subjected to sequential detergent extraction using digitonin and NP-40 as indicated. Equal aliquots from each fraction were immunoblotted using antibodies as indicated. **d**, **e** Cells were pretreated with 5 μM TAK-243 for 30 min and then exposed to 50 μM 24S-OHC for 6 h. **d** Whole-cell lysates were subjected to immunoblotting with appropriate antibodies as indicated. **e** Band intensities were quantified by densitometric scanning, relative intensity being shown. *n* = 3, **P < 0.01, when compared with cells treated with vehicle or among 24S-OHC-treated groups

To further examine 24S-OHC-induced ER stress in SH-SY5Y cells, we carried out morphological analysis using electron microscopy. To investigate changes in the ER structure during the early stages of 24S-OHC-induced cell death, cells were treated with 50 μM 24S-OHC for 3 h. In contrast to the typical rough ER structures observed in EtOH-treated control cells (Fig. 4a, arrow), we observed broken-membrane ER structures in 24S-OHC-treated cells (Fig. 4a, arrowhead). We therefore assessed whether disturbance of ER membrane integrity was induced by 24S-OHC. To do this, we employed crude subcellular fractionation to sequentially extract proteins from cells in a digitonin-soluble fraction enriched for cytosolic protein and in a subsequent NP-40-soluble fraction enriched for membrane-bound organelles including ER. The results showed that in vehicle-treated cells, the cytosolic protein DJ-1 was found in the digitonin extract; we further found that all ER proteins examined were recovered in the NP-40 extract (Fig. 4b). In cells treated with 24S-OHC for 3 h, although recovered levels of ER membrane proteins (calnexin and ribophorin-1) in NP-40 extract were unchanged, recovered levels of ER luminal proteins (GRP78, ORP150, protein disulfide isomerase (PDI) and calreticulin) were reduced in the NP-40 extract, but these proteins, except calreticulin, were increased in the DJ-1-rich digitonin extract. These changes could be inhibited by cotreatment with F12511, suggesting that ER luminal proteins were released into cytosol as a consequence of 24S-OHC esterification. We also found that levels of GRP78, ORP150, PDI, and calreticulin were increased in the digitonin extract of cells cotreated with 24S-OHC and MG132 for 6 h, indicating that these proteins were subjected to proteasomal degradation after being released into cytosol (Fig. 4c).

To investigate whether redistribution of ER luminal proteins to cytosol was the result of translocon-mediated retrotranslocation as ERAD substrates, we evaluated the effect of ubiquitination inhibition on 24S-OHC-induced chaperone degradation, because ubiquitination and retrotranslocation of luminal ERAD substrates have been shown to be coordinated by a membrane-embedded E3 ligase complex^42^. To do this, we used TAK-243, which targets the ubiquitin activating enzyme^43^, as a result of which we found that protein ubiquitination was blocked, and that the degradation of p53, a well-studied ubiquitin-dependent proteasome substrate, was inhibited by TAK-243 (Fig. 4d). We further found that cotreatment with TAK-243 did not produce the same drastic inhibition of 24S-OHC-induced chaperone protein degradation (Fig. 4e) as was observed during cotreatment with MG132 (Fig. 1g), suggesting that chaperone proteins were being released into cytosol without ubiquitination.

Collectively, these results suggest that ACAT1-mediated 24S-OHC esterification caused disruption of ER membrane integrity, resulting in the release of ER luminal proteins into cytosol, followed by ubiquitin-independent proteasomal degradation of the released proteins.

### Global protein synthesis was inhibited by 24S-OHC in SH-SY5Y cells

**Fig. 5:**
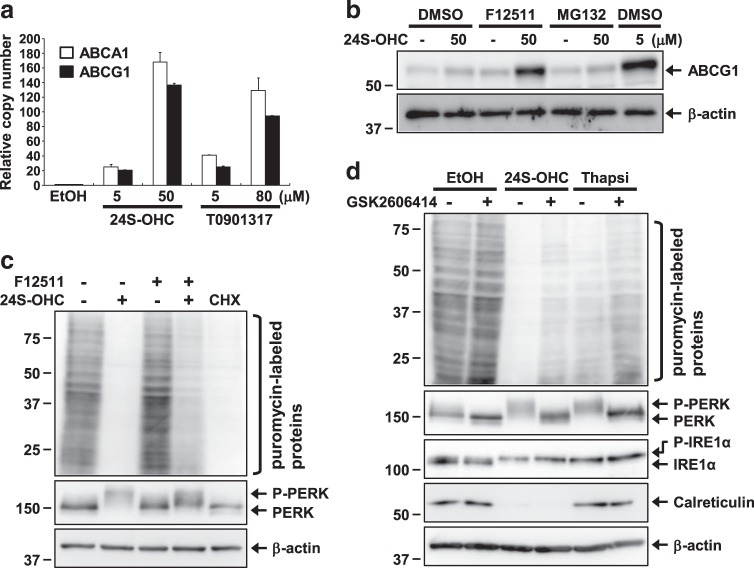
Global protein synthesis was inhibited by 24S-OHC in SH-SY5Y cells. **a** SH-SY5Y cells were treated with 24S-OHC (5 or 50 μM) or T0901317 (5 or 80 μM) for 6 h. The mRNA levels of *ABCA1* and *ABCG1* were quantified by real-time PCR. Results are shown as normalized to levels measured for *RPL32*. **b** Cells were pretreated with 5 μM F12511 or 5 μM MG132 for 30 min and then exposed to 5 or 50 μM 24S-OHC for 6 h. Whole-cell lysates were immunoblotted with antibodies specific for ABCG1 or β-actin. **c** Cells were pretreated with or without 5 μM F12511 for 15 min and then exposed to 50 μM 24S-OHC or 10 μg/ml CHX for 6 h. **d** Cells were pretreated with 5 μM GSK2606414 for 30 min and then exposed to 50 μM 24S-OHC or 3 μM thapsigargin for 6 h. **c**, **d** Cells were then incubated with 10 μg/ml puromycin for last 15 min. Whole-cell lysates were immunoblotted with antibodies specific for puromycin, PERK, IRE1α, calreticulin, or β-actin

We further examined whether 24S-OHC-induced disruption of ER membrane integrity affected de novo protein synthesis. Because 24S-OHC is known to act as a ligand of liver X receptor (LXR)^18,21^, we assessed the expression of the LXR target genes *ATP-binding cassette transporter A1 (ABCA1*) and *ABCG1*. Real-time PCR results showed that treatment for 6 h with either 24S-OHC or the synthetic LXR ligand T0901317 upregulated expression levels of *ABCA1* and *ABCG1* mRNA in a concentration-dependent manner (Fig. 5a).

Despite this observed concentration-dependent increase in *ABCG1* mRNA expression levels, however, we found that upregulation of ABCG1 protein levels was induced in cells treated with 5 μM 24S-OHC (nontoxic concentration) but not in cells treated with 50 μM 24S-OHC (Fig. 5b). We further found that cotreatment with 50 μM 24S-OHC and F12511 caused ABCG1 protein levels to increase to similar extent as was observed with 5 μM 24S-OHC, while cotreatment with 50 μM 24S-OHC and MG132 did not induce upregulation of ABCG1 protein. These results suggested that 24S-OHC esterification suppressed ABCG1 protein upregulation not as a result of proteasomal degradation, but as a result of inhibition of de novo protein synthesis.

We then evaluated the effect of 24S-OHC esterification on global protein synthesis by using SUnSET, a nonradioactive assay method utilizing incorporation of puromycin into newly synthesized proteins and subsequent detection using anti-puromycin antibody^44^. A robust decrease in puromycin-labeled proteins—not unlike that observed with the known protein synthesis inhibitor cycloheximide (CHX)—was observed in cells treated with 24S-OHC, and this decrease was found to be significantly suppressed by cotreatment with F12511 (Fig. 5c), suggesting that 24S-OHC inhibited not only ABCG1 protein synthesis but also global protein synthesis. Although 24S-OHC-induced PERK phosphorylation was completely inhibited by GSK2606414 without affecting IRE1α phosphorylation or the decrease in calreticulin levels that had been observed, the decrease in levels of puromycin-labeled proteins was partially suppressed by GSK2606414 (Fig. 5d), indicating that the observed 24S-OHC-induced robust decrease in protein synthesis was not only due to PERK-regulated translation attenuation.

## Discussion

**Fig. 6:**
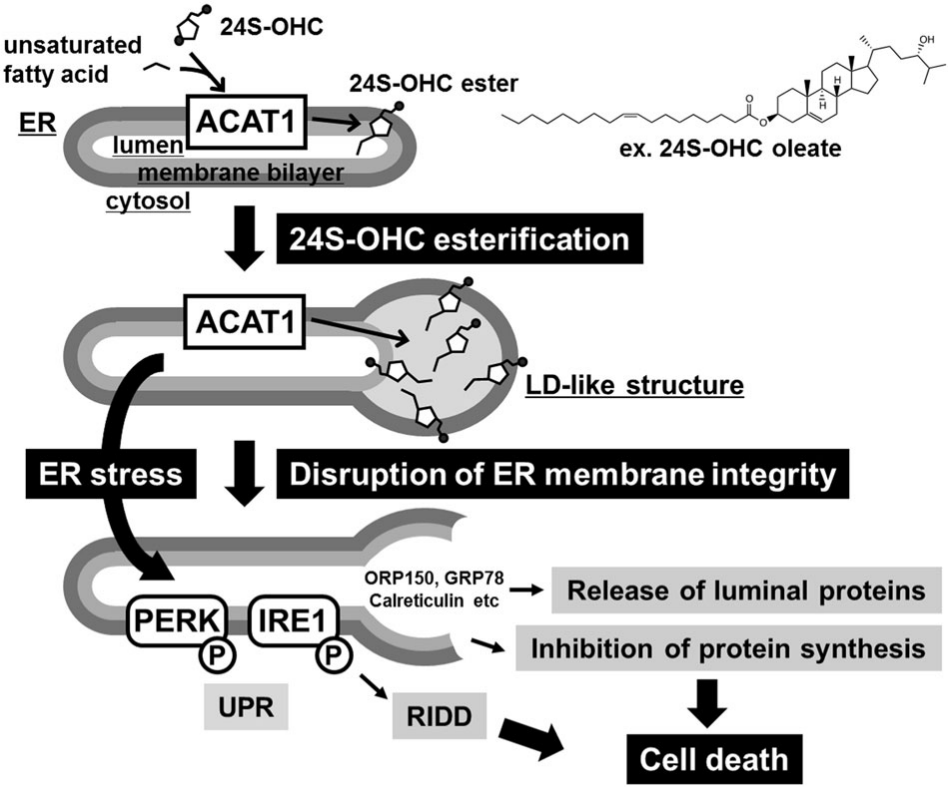
Schematic of proposed mechanism for 24S-OHC-induced cell death in SH-SY5Y cells. Under conditions of excess 24S-OHC in SH-SY5Y cells, ACAT1 catalyzes 24S-OHC ester formation from 24S-OHC and long-chain unsaturated fatty acid in the ER. Accumulation of 24S-OHC esters between the two leaflets of the ER membrane bilayer leads to formation of an LD-like structure coupled with a swollen ER structure, resulting in disruption of ER membrane integrity, which in turn induces release of ER luminal proteins. ER stress and/or disruption of ER membrane integrity activates pro-death UPR signaling including RIDD and causes inhibition of protein synthesis, triggering one unconventional type of cell death

It has been indicated that an excess of lipid can induce ER stress. For example, saturated fatty acids can activate the UPR with subsequent induction of apoptosis^13,14^. Moreover, excess cellular cholesterol can activate the UPR, resulting in induction of apoptosis in macrophages^45^. In the case of oxysterol, cytotoxic oxysterols, e.g., 7-ketocholesterol, produced by nonenzymatic auto-oxidation of cholesterol act as stressors to activate the UPR^46^. In our present study, we showed that enzymatically formed oxysterol 24S-OHC also activates the UPR signaling pathway. Regarding possible mechanisms for 24S-OHC-induced UPR activation, it is thought likely that ER dysfunction due to abnormal morphological change of ER and/or decrease in protein folding capacity causes UPR activation, since our present study demonstrates that 24S-OHC treatment results in a decrease in ER chaperone proteins. As it has been known that GRP78 binds to IRE1, PERK, and ATF6, inhibiting activation of these UPR sensors in unstressed cells, and dissociates from these UPR sensors, activating the UPR during ER stress^47^, it is also possible that reduction of luminal GRP78 levels might enforce UPR activation. Unlike the situation observed with conventional UPR, we found that 24S-OHC treatment resulted in insufficient activation of the pro-survival adaptive response, activating IRE1 and PERK but not ATF6 or XBP1. Regarding to pro-death UPR signaling, we found that RIDD plays an important role in the 24S-OHC-induced cell death machinery (Fig. 6). We postulate that since IRE1 inhibitor cannot by itself suppress 24S-OHC-induced ER membrane disruption, MKC-3946 may have some mild inhibitory effect on cell death.

Most conventional models of LD biogenesis have assumed that the neutral lipids including cholesteryl esters assemble into lens-like structures in the hydrophobic region of the ER membrane bilayer^48,49^. Once the amount of neutral lipids reaches a critical mass, the nascent LD structure buds off from the ER, followed by growth to mature LDs. Based on this model, we posit that newly formed 24S-OHC esters synthesized by ER-resident ACAT1 might be packaged between the two leaflets of the ER membrane bilayer in similar fashion as would be the case with a cholesteryl esters (Fig. 6). Accumulation of 24S-OHC esters results in the formation of LD-like structures. As identified in our previous study^33^, 24S-OHC esters have a hydroxyl group at position 24 in their steroid side chain (e.g., 24S-OHC oleate). More specifically, we postulate that because the 24S-OHC ester has the polar moiety of its side chain plus the nonpolar moiety that results from combination of its sterol ring with an unsaturated fatty acid, 24S-OHC esters may possess amphipathic properties within the hydrophobic environment of the lipid bilayer. It is therefore plausible that, unlike cholesteryl esters which are entirely hydrophobic, amphipathic properties of 24S-OHC esters could present a detergent function to the ER bilayer. Indeed, electron microscopy and biochemical analysis revealed that treatment with 24S-OHC caused ER membrane integrity to be disrupted and ER luminal proteins to be subsequently released into cytosol. The possibility that some other proteins mediate 24S-OHC-induced disruption of ER membrane integrity cannot be excluded. It was interesting to note that the released ER proteins were subjected to proteasomal degradation without ubiquitination, suggesting that ubiquitin-independent degradation by the 20 S proteasome but not ubiquitin-dependent degradation by the 26 S proteasome might be responsible for degradation of the released proteins. We also found that not only ER luminal proteins but also some ER transmembrane proteins such as calnexin and ATF6 were degraded by proteasome during 24S-OHC-induced ER stress. Further studies are needed to clarify the biological mechanism for 24S-OHC-induced proteasomal degradation of ER proteins.

We also demonstrated that de novo synthesis of global protein was robustly suppressed in 24S-OHC-treated cells, and that the observed decrease in protein synthesis was only partially recovered by PERK inhibitor, suggesting that not only PERK-induced translational attenuation but also other mechanisms might be involved. Since it is well-established that global protein synthesis inhibition induces cell death, it is thought that the observed 24S-OHC-induced robust decrease in de novo protein synthesis may play an important role in the cell death machinery. Because we found that mRNA levels of LXR target genes were markedly upregulated in 50 μM 24S-OHC-treated cells, it is unlikely that the observed robust decrease in nascent proteins was due to reduction in global transcription activity. Degradation of ER-localized mRNAs by RIDD might be partially implicated in the observed decrease in nascent proteins. Because 24S-OHC treatment caused disruption of ER membrane integrity, it is therefore plausible that protein synthesis was downregulated due to disappearance of the scaffold for protein translation at the rough ER. There is a possibility that the weak induction levels of XBP1s, ATF4, and CHOP proteins we observed might be associated with the 24S-OHC-induced robust decrease in protein synthesis.

A growing body of experimental evidence has unveiled and characterized the existence of multiple types of cell death^29^. In addition to apoptosis and necrosis, many nonapoptotic regulated cell death (RCD) pathways are emerging, including necroptosis (regulated necrosis), pyroptosis, parthanatos, ferroptosis, and several others. Furthermore, novel signaling pathways thought to orchestrate RCD are still being characterized. While multiple types of RCD share common morphological hallmarks and are regulated by overlapping and interconnected signaling pathways, the various modes of RCD are distinguishable to a certain degree based on features observed or inferred to exist during the various stages of the respective cell death processes^29,50^. We have previously demonstrated that SH-SY5Y cells treated with 24S-OHC exhibited neither fragmentation of the nucleus nor caspase activation^28^. We have also found that 24S-OHC-treated cells showed morphological changes resembling necrosis, but these did not induce ATP depletion^28^. We might summarize the results of our current and previous findings by saying that the mode of 24S-OHC-induced cell death in SH-SY5Y cells is characterized by: (i) ACAT-mediated 24S-OHC esterification-dependency; (ii) activation of pro-death UPR signaling including RIDD; (iii) disruption of ER membrane integrity; and (iv) inhibition of protein synthesis (Fig. 6). Based on this unique combination of features, we conclude that 24S-OHC-induced cell death might be an unconventional type of RCDs. Since it has been reported that RIPK1 is a mediator of ER stress-induced apoptosis^51^, the dependency with respect to RIPK1 suggests a possible connection with the downstream branch of 24S-OHC-induced cell death signaling. Moreover, we have shown that accumulation of 24S-OHC esters induces caspase-dependent apoptotic cell death in Jurkat cells or all-*trans*-retinoic acid-treated SH-SY5Y cells in which caspase-8 is expressed^32,52^, suggesting that the executor(s) in the downstream pathway(s) of 24S-OHC-induced cell death may exhibit cell-type dependency. In studies with other oxysterols, we previously showed that 7-ketocholesterol, 7α-OHC, 7β-OHC, and 22R-OHC induced cell death in SH-SY5Y cells, and that this was not suppressed by ACAT inhibitor^32^. On the other hand, ACAT is implicated in 24(S),25-epoxycholesterol-induced apoptosis in mast cells^53^, and in 7-ketocholesterol-induced apoptosis in macrophages^54^. Since growing evidence suggests a link between accumulation of oxysterols and the pathophysiologies of various diseases^55,56^, further investigations will be required to address the features and specificities of 24S-OHC-induced cell death in oxysterol-induced cell death. In conclusion, our present study revealed that abnormal ER morphological change with disruption of ER membrane integrity is crucial for triggering of pro-death signaling under conditions in which there is accumulation of 24S-OHC esters by ACAT1.

## Materials and methods

### Materials

24S-OHC was synthesized as we previously reported^57^, this have been dissolved in EtOH (Wako, Osaka, Japan) and stored at −20 °C. F12511 ACAT inhibitor was the generous gift of Dr. Ta-Yuan Chang (Geisel School of Medicine at Dartmouth, Hanover, NH, USA). Thapsigargin, MG132, and CHX were purchased from Wako (Osaka, Japan). Nec-1, SP600125, and 4-PBA were from Sigma-Aldrich (St. Louis, MO, USA). 4μ8C and MKC-3946 were from Merck Millipore (Darmstadt, Germany). GSK2606414, T0901317, NQDI-1, and SB203580 were from Cayman Chemical (Ann Arbor, MI, USA). TAK-243 was from Active Biochem (Hong Kong, China). Thapsigargin, MG132, Nec-1, GSK2606414, 4μ8 C, T0901317, TAK-243, CHX, NQDI-1, SB203580, and SP600125 were dissolved in dimethyl sulfoxide (DMSO, Wako). 4-PBA was dissolved in 1 M NaOH. The following antibodies were from commercial sources: IRE1αα (Cat# 3294), PERK (Cat# 3192), ATF4 (Cat# 11815), and CHOP (Cat# 2895) were all from Cell Signaling (Danvers, MA, USA); ATF6α (Cat# 73500) was from BioAcademia (Osaka, Japan); β-actin (Cat# A5441) was from Sigma-Aldrich. XBP1 (Cat# 24168-1-AP) was from Proteintech (Chicago, IL, USA); GRP78 (Cat# 610979), calreticulin (Cat# 612136), calnexin (Cat# 610523), and (PDI Cat# 610946) were from BD Biosciences (Franklin Lakes, NJ, USA); ORP150 (Cat# 10301) was from IBL (Gunma, Japan); ribophorin 1 (Cat# sc-12614), ubiquitin (Cat# sc-8017), and DJ-1 (Cat# ab4150) were from Abcam (Cambridge, UK); p53 (Cat# OP43) and puromycin (Cat# MABE343) were from Merck Millipore; ABCG1 (Cat# NB400-132) was from Novus Biologicals (Littleton, CO, USA). All other chemicals, of analytical grade, were obtained from Sigma-Aldrich or Wako.

### Cell lines and cell culture

Human neuroblastoma cells from the SH-SY5Y cell line were purchased from American Type Culture Collection (Manassas, VA, USA) or European Collection of Cell Cultures (Salisbury, UK). Cells were routinely maintained in the Dulbecco’s Modified Eagle’s Medium/Nutrient Mixture Ham’s F-12 (Thermo Fisher Scientific, Waltham, MA, USA), which contained 10% heat-inactivated fetal bovine serum (Sigma-Aldrich), and antibiotics (100 U/ml penicillin, 100 μg/ml streptomycin; Thermo Fisher Scientific). Cells were grown at 37 °C under an atmosphere of 95% air and 5% CO_2_.

### Cell treatment

To elucidate the mechanism of 24S-OHC-induced cell death, SH-SY5Y cells were treated with 50 μM 24S-OHC for the indicated period (final concentration of EtOH in the medium was 0.5%). Cells were also exposed to 3 μM thapsigargin for the indicated period as positive controls. EtOH or DMSO was used as a vehicle for control treatments. To evaluate the effect of various inhibitors, cells were pretreated with 5 μM F12511 for 15 min, or with 20 μM MG132, or with 5 μM TAK-243 for 30 min, before further treatment. Cells were also pretreated with variable concentrations of GSK2606414, 4μ8 C, MKC-3946, NQDI-1, SB203580, or SP600125 for 1 h, or 4-PBA for 24 h, before further treatment.

### Immunoblotting

Whole-cell extract was prepared as described previously^58^. Briefly, cells were suspended in lysis buffer (150 mM NaCl, 50 mM Tris-HCl at pH 7.5, 1% NP-40, 2 mM EDTA) containing PhosSTOP (Roche Applied Science, Mannheim, Germany) and a protease inhibitor cocktail (Nacalai Tesque, Kyoto, Japan) at 4 °C for 30 min. Nuclei and unlysed cellular debris were removed by centrifugation at 13,000×*g* for 5 min. Protein samples were subjected to SDS–PAGE and were transferred to a PVDF membrane for 1 h at 100 V. Immunoblotting with appropriate antibodies was visualized with enhanced chemiluminescence (Millipore, Billerica, MA, USA).

### *XBP1*-splicing assay

The total RNA isolation and real-time PCR were conducted as described previously^20^. Briefly, the total RNA was isolated by using Tripure Isolation Reagent (Roche Applied Science) along with chloroform according to the manufacturer’s instructions. Synthesis of cDNA was carried out using a PrimeScript^TM^ RT Master Mix (Takara Bio, Shiga, Japan) according to the manufacturer’s instruction. The *XBP1*-splicing assay used human *XBP1*-specific primers that amplified and spliced (-26 nt) and unspliced *XBP1* mRNA (forward 5'-TTACGAGAGAAAACTCATGGCC-3', reverse 5'-GGGTCCAAGTTGTCCAGAATGC-3'). PCR products were analyzed on a 3% agarose gel.

### Determination of cell viability

For determination of cell viability, WST-8 assay and lactate dehydrogenase (LDH) assay were used. WST-8 assay was performed using Cell Counting Kit-8 according to the manufacturer’s instructions (Dojindo, Kumamoto, Japan). LDH activity assay was performed as described previously^30^. The data are expressed as the percentage of total LDH activity, after subtraction of background determined from the culture medium alone.

### Knockdown of CHOP by small interfering RNA

Double-stranded small interfering RNAs (siRNA) targeting human *CHOP* were obtained from Thermo Fisher Scientific. The sense and antisense of siRNA specific for CHOP used here were as follows: #1: 5'-UAGCUGAAGAGAAUGAACGGCUCAA-3' and 5'-UUGAGCCGUUCAUUCUCUUCAGCUA-3'; #2: 5'-GCAAGAGGUCCUGUCUUCAGAUGAA-3' and 5'-UUCAUCUGAAGACAGGACCUCUUGC-3'; #3: 5'-UAGAGGCGACUCGCCGAGCUCUGAU-3' and 5'-AUCAGAGCUCGGCGAGUCGCCUCUA-3'. Stealth RNAi-negative control (NC) with medium GC content (48%) was obtained from Thermo Fisher Scientific. The siRNAs were transfected into SH-SY5Y cells at a concentration of 100pmol/well by Lipofectamine RNAiMAX (Thermo Fisher Scientific) for 24 h before further experiments. To confirm knockdown efficiency for CHOP, expression levels of protein were analyzed by immunoblotting analysis.

### Real-time PCR

Quantitative detection of differentially expressed genes was performed with the 7900HT Fast Real-Time PCR system (Applied Biosystems, Foster City, CA, USA) and SYBR Green Master Mix (Thermo Fisher Scientific) under the following condition: one cycle of 95 °C for 10 min followed by 40 cycles of 95 °C for 15 s and 60 °C for 1 min. Human *RPL32* (ribosomal protein L32) was used for the normalization of each reaction. The following primers (Thermo Fisher Scientific) were used: human *ANGPTL3* forward 5'-AACATGATGGCATTCCTGCTGA-3', reverse 5'-GAGTTGCTGGGTCTGATGGCA-3'; human *BLOS1* forward 5’-GAGGAGGCGAGAGGCTATCA-3', reverse 5'-ATCCCCAATTTCCTTGAGTGC-3'; human *COL6* forward 5’-CAACGACATTGCACCCCGAG-3', reverse 5'-CCGCACTTGCATTCACAGCA-3'; human *PDGFRB* forward 5’-CAGCAAGGACACCATGCGGC-3', reverse 5'-TGGGACATCCGTTCCCACAC-3'; human *SCARA3* forward 5’-ACGAGGACATGCCGACCTTC-3', reverse 5'-TTCAGGGCTTTCGGATCCAGG-3'; human *RPL32* forward 5’-CCCCTTGTGAAGCCCAAGA-3', reverse 5'-TGACTGGTGCCGGATGAAC-3'.

### Electron microscopy analysis

For electron microscopy, ultrathin sections were prepared as described previously^33^, and were subjected to TEM observation (JEM-1200 EX, JEOL) at Hanaichi Ultrastructure Research Institute (Okazaki, Japan).

### Subcellular fractionation

Crude cytosolic and membrane/organelle fractions were isolated by sequential detergent extraction as described previously^59,60^. Equal aliquots from each fractions were analyzed for immunoblotting.

### Measurement of protein synthesis by SUnSET puromycin end-labeling assay

Following treatment, cells were incubated with 10 μg/ml puromycin for 15 min prior to cell lysis. Whole-cell lysates were then subjected to immunoblotting. An anti-puromycin antibody was used to detect levels of puromycin-labeled proteins.

### Statistical analysis

The data are reported as mean ± SD of at least three independent experiments unless otherwise indicated. The statistical significance of the difference between the determinations was calculated by analysis of variance using ANOVA, Tukey–Kramer multiple comparisons test. The difference was considered significant when the *P*-value was <0.05.

## Supplementary information

Supplemental Figure 1-3
