# Supplementary material for: 24(S)-Hydroxycholesterol induces ER dysfunction-mediated unconventional cell death
Source: Cell Death Discov. 2019 Jul 5;5:113. doi: 10.1038/s41420-019-0192-4 (PMC6611791; doi:10.1038/s41420-019-0192-4)
Supplement: Supplementary file 1 — Supplemental Figure 1-3 [file 41420_2019_192_MOESM1_ESM.pdf]

Figure S1

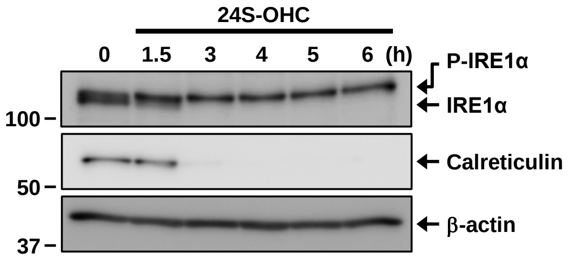

Figure S1. Phosphorylation of IRE1α and decrease in calreticulin occurred substantially simultaneously in SH-SY5Y cells. SH-SY5Y cells were treated with 50 μM 24S-OHC for 1.5-6 h. Whole cell lysates were immunoblotted with antibodies specific for IRE1α, calreticulin, or β-actin.

Figure S2

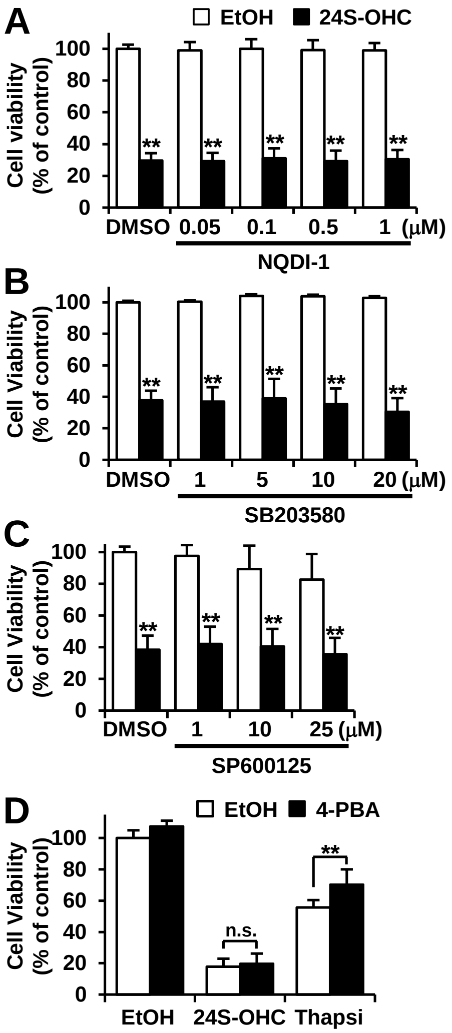

Figure S2. 24S-OHC-induced cell death in SH-SY5Y cells was not suppressed by 4-PBA or by inhibitors specific for ASK1, p38, or JNK. (A-C) SH-SY5Y cells were pretreated with variable concentrations of NQDI-1 (A), SB203580 (B), or SP600125 (C) for 1 h and then exposed to 50 μM 24S-OHC for 24 h. **P < 0.01, when compared with cells treated with vehicle. (D) Cells were pretreated with 1 mM 4-PBA for 24 h and then exposed to 50 μM 24S-OHC or 3 μM thapsigargin for 24 h. Cell viability was measured by WST-8 assay. **P < 0.01.

Figure S3

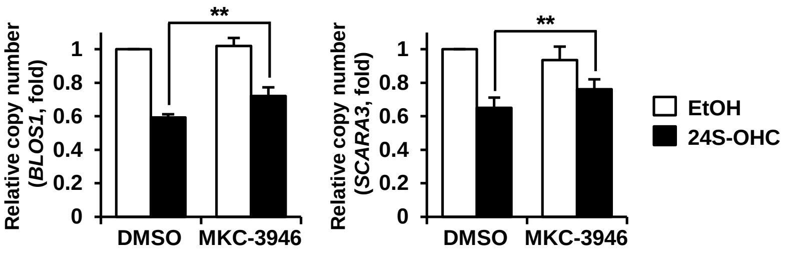

Figure S3. MKC-3946 inhibited 24S-OHC-induced down-regulation of *BLOS1* and *SCARA3* mRNA expression. Cells were pretreated with 7.5 μM MKC-3946 for 1 h then exposed to 50 μM 24S-OHC for 6 h. The mRNA levels of *BLOS1* and *SCARA3* were quantified by real-time PCR. **P < 0.01, when compared with cells treated with 24S-OHC alone.
